# Endovascular treatment of transplant renal artery stenosis based on hemodynamic assessment using a pressure wire: a case report

**DOI:** 10.1186/s12872-018-0909-y

**Published:** 2018-08-22

**Authors:** Yoshito Kadoya, Kan Zen, Satoaki Matoba

**Affiliations:** Department of Cardiovascular Medicine, Graduate School of Medical Science, Kyoto Prefectural University of Medicine, 465 Kajii-cho, Kawaramachi-Hirokoji, Kamigyo-ku, Kyoto, 602-8566 Japan

**Keywords:** Endovascular treatment, Transplant renal artery stenosis, Case report

## Abstract

**Background:**

Transplant renal artery stenosis (TRAS) is a serious complication after renal transplantation, leading to hypertension, deterioration in renal function, and/or graft loss. The incidence of TRAS reportedly varies from 1 to 23%, depending on its definition or diagnostic tools. The hemodynamic definition or therapeutic indication of TRAS is unclear.

**Case presentation:**

A 66-year-old man with a history of diabetes, chronic kidney disease, and angina presented with a 2-week history of dyspnea and leg edema. He had undergone living-donor kidney transplantation for end-stage renal disease secondary to diabetic nephropathy 7 years earlier. He developed acute deterioration in renal function after the administration of an angiotensin II receptor blocker and required emergency hospitalization owing to acute congestive heart failure with pulmonary edema. A vasodilator and loop diuretics were administered following his admission, and the patient’s symptoms resolved quickly. Further investigation, including magnetic resonance angiography and ultrasonography, revealed severe stenosis of the transplant renal artery. Renal arteriography and pressure gradient measurement using a 0.014-inch pressure wire were performed. The systolic pressure gradient was 40 mmHg, and the resting Pd/Pa ratio (ratio of mean distal to lesion and mean proximal pressures) was 0.90 without hyperemia. Hemodynamically significant stenosis was suspected. Intravascular ultrasonography revealed vessel shrinkage in the stenotic area, suggestive of the end-to-end anastomosis site. Pre-dilation using a 4-mm balloon, implantation of a 6-mm self-expandable stent, and post-dilatation using a 5-mm balloon were performed. Although the moderate stenosis persisted angiographically, the systolic pressure gradient dropped to 20 mmHg with the mean systolic pressure ratio increased to 0.95, which was considered an acceptable result. One month after the procedure, the patient’s renal function and blood pressure control had significantly improved.

**Conclusions:**

Hemodynamic assessment using a pressure wire is useful in determining the appropriate therapeutic indication and endpoint of endovascular treatment of TRAS.

## Background

Transplant renal artery stenosis (TRAS) is a serious complication after renal transplantation, and is an important cause of hypertension, renal function deterioration, and/or graft loss [1]. The reported incidence of TRAS is in the range of 1–23%, depending on its definition or on the diagnostic tools [2]. Endovascular treatment (EVT), including percutaneous transluminal angioplasty and stent implantation, is considered the first-line therapy for TRAS [3]. However, the hemodynamic definition or therapeutic indication of TRAS remains unclear. Herein, we report a case of TRAS that was successfully treated via EVT based on hemodynamic assessment using a 0.014-inch pressure wire.

## Case presentation

**Fig. 1:**
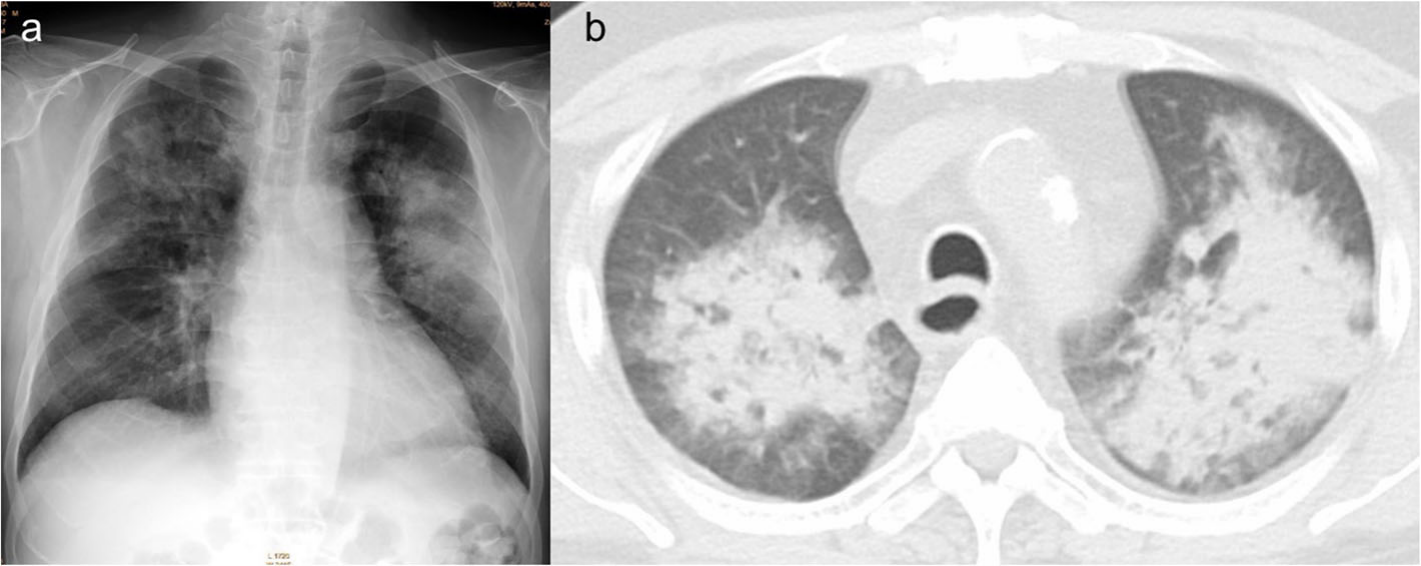
**a** Plain chest radiography showing a bilateral infiltrative shadow. **b** Computed tomography of the chest showing a bilateral infiltrative shadow in the hilar region dominant

**Fig. 2:**
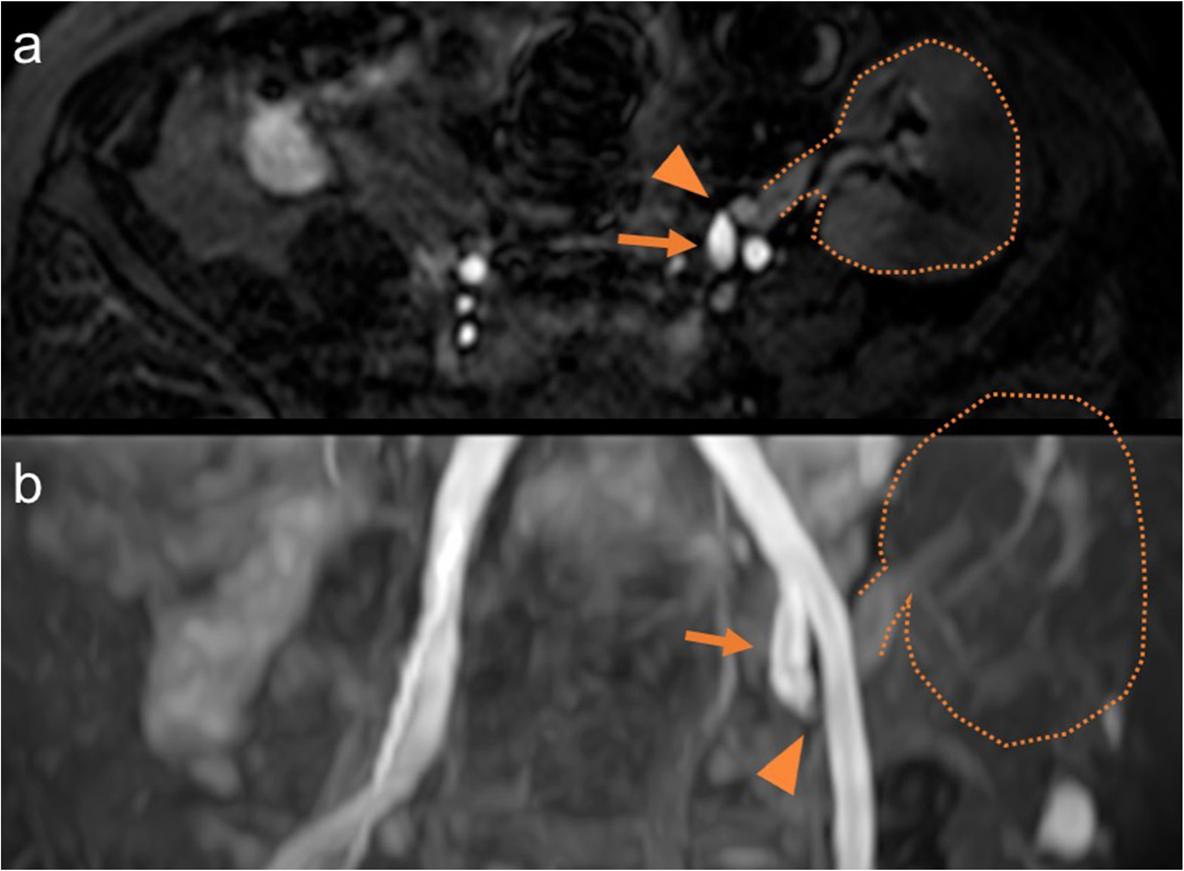
Magnetic resonance angiography showing the bending and narrowing of the transplant renal artery (**a** axial view; **b** three-dimensional image) (arrow: the left internal iliac artery; arrowhead, the narrowing part; dash line: the transplanted kidney)

**Fig. 3:**
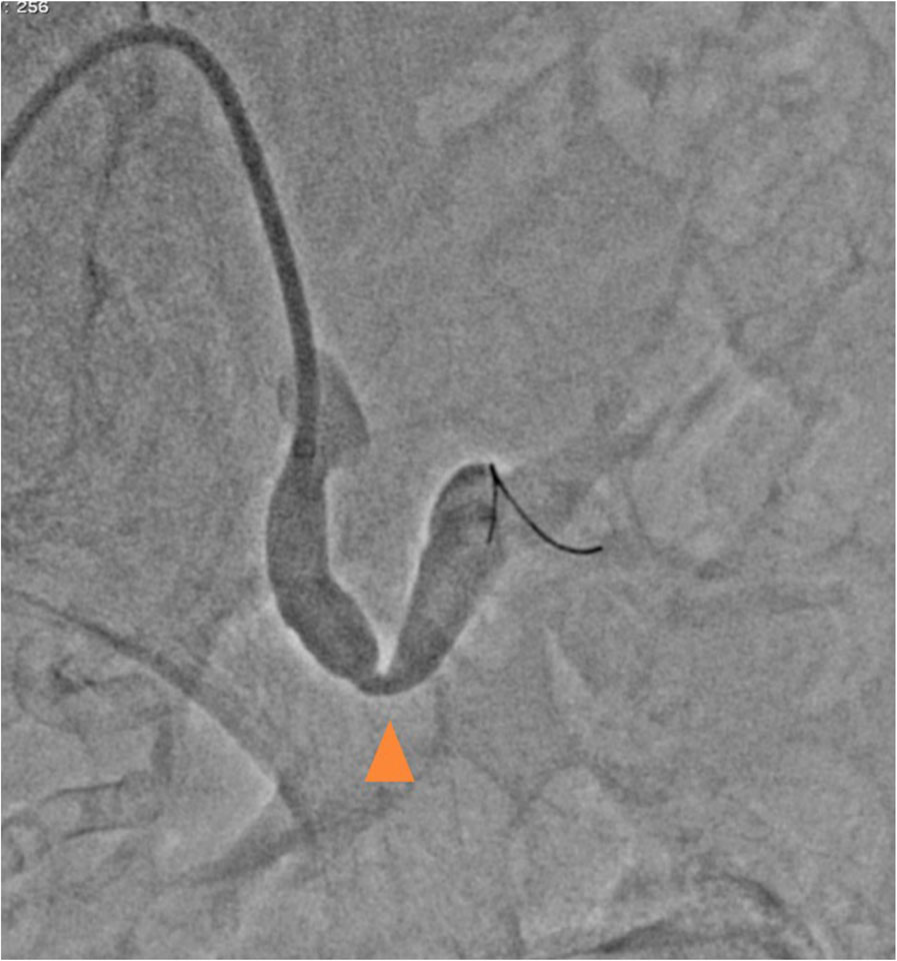
Angiography showing significant stenosis in the transplant renal artery (arrowhead: the narrowing part)

A 66-year-old Japanese man with a history of diabetes, chronic kidney disease, and angina was admitted to our hospital with a 2-week history of dyspnea and leg edema. He also had a history of end-stage renal failure secondary to diabetic nephropathy and had been undergoing peritoneal dialysis. He had received living-donor kidney transplantation from his wife 7 years earlier, in which an end-to-end anastomosis of the donor renal artery to the patient’s left internal iliac artery was performed. After the transplantation, he was able to discontinue dialysis and his renal function was stable with an estimated glomerular filtration rate (eGFR) of approximately 40 mL/min/1.73 m^2^. A few months before admission to our hospital, his blood pressure control gradually deteriorated, and he experienced acute deterioration in renal function after the administration of an angiotensin II receptor blocker (ARB). Antihypertensive medication on admission included 2.5 mg of carvedilol, 80 mg of nifedipine, and 2 mg of benidipine. On physical examination, he exhibited wheezing in the chest and pitting edema in the bilateral limbs. His body temperature was 36.8 °C; blood pressure, 166/71 mmHg; regular pulse rate, 91 beats/min; and oxygen saturation, 93% (without oxygen administration). Laboratory findings showed acute exacerbation of renal function with an eGFR of 24 mL/min/1.73 m^2^ and an elevation of the brain natriuretic peptide level (483.3 pg/mL; normal range: < 18.4 pg/mL). Electrocardiography showed the strain pattern. Echocardiography revealed concentric left ventricular hypertrophy as well as moderate aortic stenosis with an aortic mean gradient of 11 mmHg, a valve area of 1.12 cm^2^, and an ejection fraction of 68%. The severity of aortic stenosis had been followed up echocardiographically once yearly, showing no significant progression at this hospitalization. Plain chest radiography and computed tomography of the chest showed a bilateral infiltrative shadow, suggestive of pulmonary edema (Fig. 1). The patient’s diagnosis was acute congestive heart failure with pulmonary edema. A vasodilator and loop diuretics were administered following admission, and the patient’s symptoms resolved quickly. Because of the worsened blood pressure control and renal function deterioration caused by ARB, transplant renal artery stenosis was suspected. Magnetic resonance angiography was performed and it revealed the bending and narrowing of the transplant renal artery (Fig. 2). On ultrasound examination, the peak systolic velocity of the narrowing part was observed to be above 2 m/sec. The peak systolic velocity ratio could not be determined owing to poor echographic imaging of the proximal non-stenosed part. A 5-French guiding catheter (Destination®, Terumo, Japan) was inserted into the left common iliac artery via the right femoral artery and an angiography revealed significant stenosis in the transplant renal artery (Fig. 3).

**Fig. 4:**
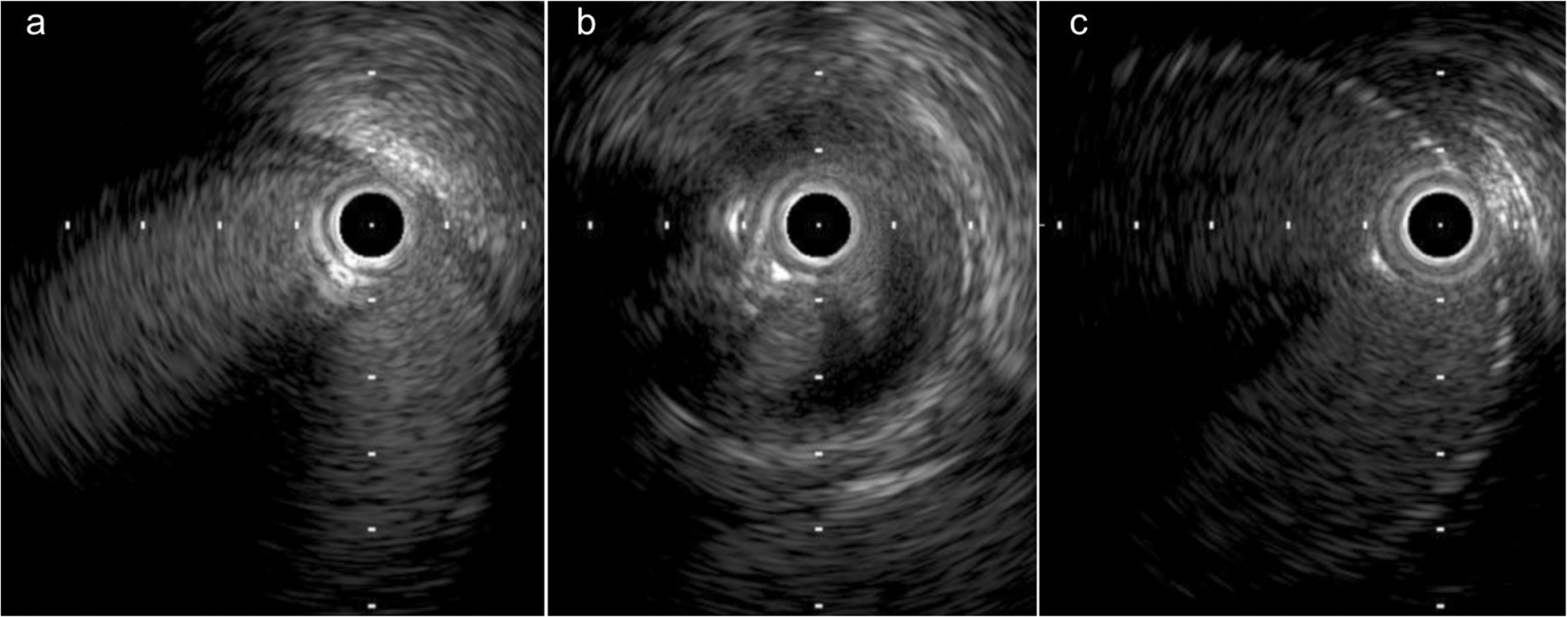
Intravascular ultrasonography showing vessel shrinkage at the narrowing part, with a diameter of 5 mm (**a** proximal part; **b** the narrowing part; **c** distal part)

**Fig. 5:**
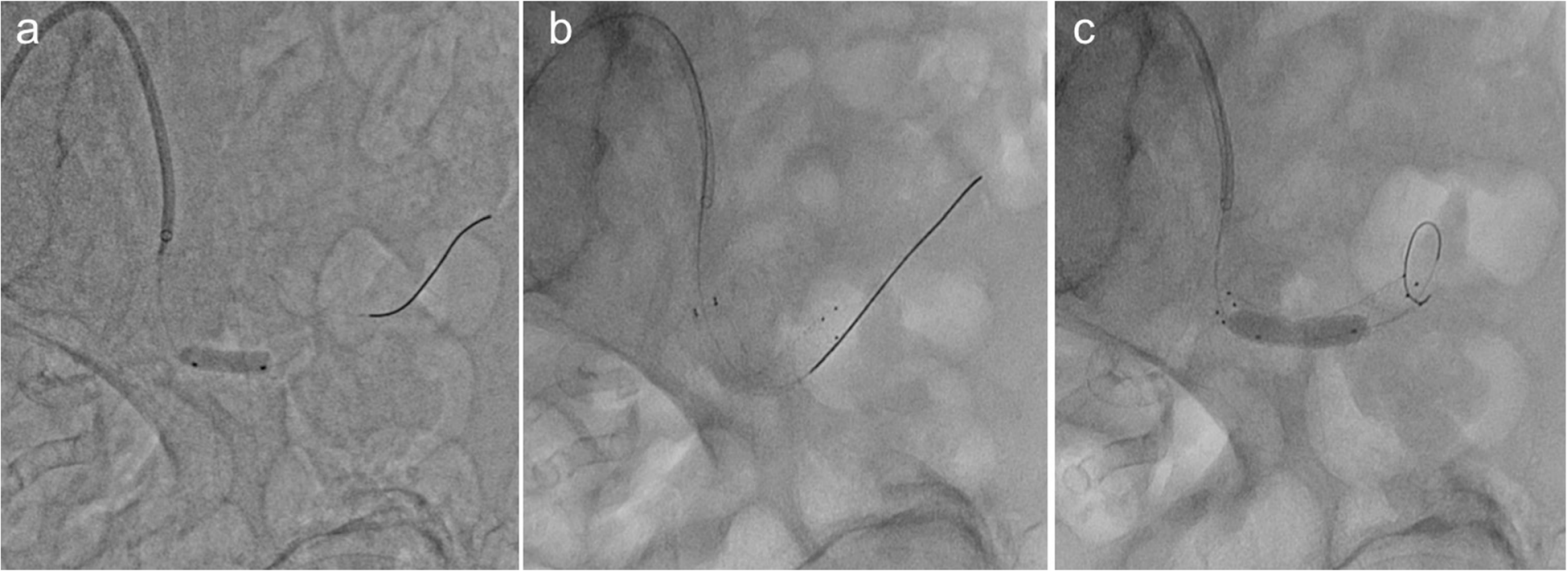
Procedure of endovascular treatment. **a** Pre-dilation using a 4-mm balloon. **b** Implantation of a 6-mm self-expandable stent. **c** Post-dilatation using a 5-mm balloon

**Fig. 6:**
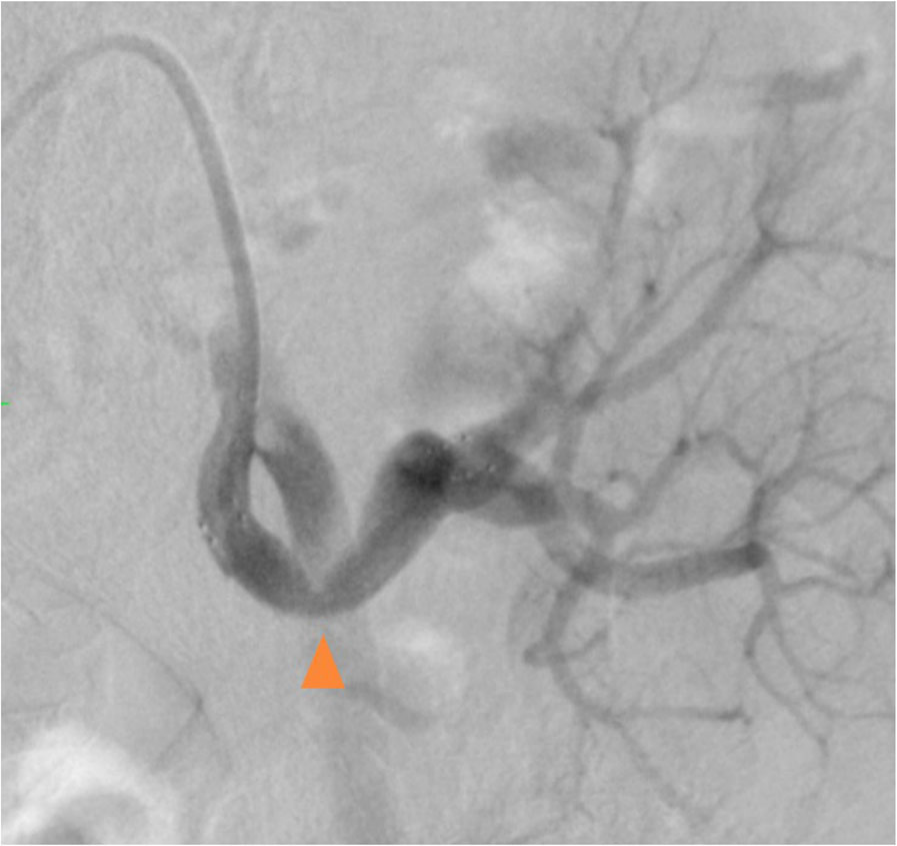
Final angiography showing persistent moderate stenosis (arrowhead)

In addition, pressure gradient measurement was conducted using a 0.014-inch pressure wire (Aeris®, Abbott, USA). The mean pressure was 96 mmHg proximally and 87 mmHg distally with a resting Pd/Pa ratio (ratio of mean distal to lesion and mean proximal pressures) of 0.90, and the peak-to-peak systolic pressure gradient was 40 mmHg without hyperemia. Based on these findings in addition to the clinical course, such as worsening of blood pressure control and acute renal function deterioration after the administration of an ARB, we assessed that the stenosis was hemodynamically significant. Therefore, we did not perform hyperemic evaluation of the stenosis. Intravascular ultrasonography (IVUS) revealed shrinkage of the vessel in the stenotic area, with a diameter of 5 mm, suggestive of the anastomosis site (Fig. 4). Pre-dilation using a 4-mm balloon, implantation of a 6-mm self-expandable stent, and post-dilatation using a 5-mm balloon were sequentially performed (Fig. 5). Thereafter, moderate stenosis persisted angiographically (Fig. 6). However, we had concerns that too much dilatation would increase the risk of vessel dissection or perforation because the lesion might be at the anastomosis site. We repeated the resting pressure gradient measurement. The mean pressure proximally was 101 mmHg and the pressure distally was 96 mmHg, with a resting Pd/Pa ratio of 0.95, and the peak-to-peak systolic pressure gradient was 20 mmHg. This was thought to be acceptable as the endpoint of TRAS-EVT. Moreover, the self-expandable stent was expected to further expand in the remote phase. Therefore, we concluded the procedure. After the procedure, the peak systolic velocity in the stenosed part based on an ultrasound examination had dropped to 1.45 m/sec. Renal function and blood pressure control improved, which resulted in the preservation of graft function. Moreover, antihypertensive medication could be significantly reduced; carvedilol 1.25 mg and nifedipine 40 mg. Four months later, a follow-up angiography demonstrated no restenosis and the pressure gradient had dropped to 15 mmHg.

## Discussion and conclusions

We present a case of TRAS, which was successfully treated via EVT based on a hemodynamic assessment using a pressure wire. To the best of our knowledge, this is the first reported case of pressure wire-guided EVT of TRAS. Hemodynamic assessment should be conducted to determine the therapeutic indication and endpoint of EVT for TRAS.

The reported incidence of TRAS in the literature varies from 1 to 23%, being an important cause of hypertension, renal function deterioration, and/or graft loss (1). The wide range of TRAS incidence may be influenced by the non-standard definition of the hemodynamic significance of TRAS [2, 3]. Marini et al. defined hemodynamically significant TRAS as > 75% narrowing of the lumen diameter [2]. On the other hand, Nasserala et al. defined it as > 50% narrowing of the arterial lumen [4]. Although the gold standard for the diagnosis of TRAS remains renal arteriography, angiographic stenosis was reported to have a poor correlation with hemodynamic parameters [5]. Doppler ultrasonography might provide hemodynamic information; however, such data cannot be obtained accurately owing to patients’ anatomical factors, such as obesity or vessel angulation. Invasive pressure measurement using 4- or 5-French catheters during angiography also seems inappropriate because it can overestimate the true pressure gradient due to the flow obstruction [6, 7]. Thus, we thought pressure gradient measurement using a 0.014-inch pressure wire might be a more useful and appropriate method for the assessment of hemodynamic function in TRAS.

In recent years, the concept of renal fractional flow reserve (FFR) using a pressure wire has been developed. Renal FFR is calculated as the ratio of the mean distal renal pressure to the mean aortic pressure during hyperemia. Intrarenal papaverine is commonly used for eliciting maximal renal hyperemia. Although renal FFR threshold still remains controversial regarding the clinical benefit of revascularization in a patient with renovascular hypertension [8, 9], Leesar MA et al. showed that a hyperemic systolic gradient ≥21 mmHg is the predictor of hypertension improvement after stenting of renal artery stenosis [10]. Kapoor N et al. suggested that a hyperemic systolic gradient ≥21 mmHg or a renal FFR of 0.90 can be considered a hemodynamically significant stenosis, and resting systolic gradient would underestimate the significance of stenosis [11]. In the present case, resting pressure measurement using 0.014-inch wire revealed a systolic pressure gradient of 40 mmHg and a Pd/Pa ratio of 0.90; therefore, we assessed the lesion as a hemodynamically significant stenosis. We considered that hyperemic evaluation would not be mandatory when it was clear that the lesion was a significant stenosis based on resting hemodynamic evaluation. To evaluate the indication of TRAS treatment, the patients’ clinical course, such as worsening of blood pressure control and acute renal function deterioration, is undoubtedly important. In addition, we believe the hemodynamic evaluation with or without hyperemia adds useful information to confirm the indication of TRAS treatment.

Using a 0.014-in. wire during renal arteriography offers some technical advantages to TRAS-EVT. Regarding the treatment strategy, EVT is recognized as the first-line therapy for TRAS; its technical success is reported in 89–100% of cases [12]. After pressure measurement using a 0.014-in. wire, the subsequent EVT procedure can be performed and completed using the same wire. Moreover, pressure measurement seems to be useful to determine the endpoint of EVT. We thought that angiographically complete dilation may increase the risk of vessel dissection or perforation; and is not required for the maintenance of graft function. In the present case, the patients’ renal function and blood pressure control sufficiently improved despite the persistent moderate stenosis. Therefore, it is important to identify the appropriate endpoints and avoid serious complications induced by excessive dilatation.

Recently, hemodynamic evaluation using a pressure wire has become quite popular in cardiac catheterization. This technology should be used more widely in various peripheral artery interventions, including lower limb arteries or renal arteries. It is essential to clarify the correct therapeutic indication and endpoint of EVT in non-coronary locations based on the hemodynamic evaluation with or without hyperemia. Although the possible limitation of hyperemia achievement in chronic kidney disease patients was reported [13], coronary circulation and other peripheral circulations should not be considered in the same way, and we think further studies are necessary.

In conclusion, we described a case of TRAS that was successfully treated via EVT based on pressure gradient measurement using a pressure wire. Hemodynamic evaluation using a pressure wire is useful in determining the therapeutic indication and endpoint of EVT in TRAS treatment.

## Abbreviations

ARB: Angiotensin II receptor blocker
eGFR: Estimated glomerular filtration rate
EVT: Endovascular treatment
FFR: Fractional flow reserve
IVUS: Intravascular ultrasound
TRAS: Transplant renal artery stenosis
